# Experiences from ten years of incident reporting in health care: a qualitative study among department managers and coordinators

**DOI:** 10.1186/s12913-018-2876-5

**Published:** 2018-02-14

**Authors:** Siw Carlfjord, Annica Öhrn, Anna Gunnarsson

**Affiliations:** 1 Department of Medical and Health Sciences, Division of Community Medicine, Linköping University, SE-58183 Linköping, Sweden; 2 Centre for Healthcare Development, County Council of Östergötland, SE-581 91 Linköping, Sweden; 3 Department of Emergency Medicine and Department of Clinical and Experimental Medicine, Linköping University, SE-58183 Linköping, Sweden

**Keywords:** Patient safety, Incident reporting, Qualitative research

## Abstract

**Background:**

Incident reporting (IR) in health care has been advocated as a means to improve patient safety. The purpose of IR is to identify safety hazards and develop interventions to mitigate these hazards in order to reduce harm in health care. Using qualitative methods is a way to reveal how IR is used and perceived in health care practice. The aim of the present study was to explore the experiences of IR from two different perspectives, including heads of departments and IR coordinators, to better understand how they value the practice and their thoughts regarding future application.

**Methods:**

Data collection was performed in Östergötland County, Sweden, where an electronic IR system was implemented in 2004, and the authorities explicitly have advocated IR from that date. A purposive sample of nine heads of departments from three hospitals were interviewed, and two focus group discussions with IR coordinators took place. Data were analysed using qualitative content analysis.

**Results:**

Two main themes emerged from the data: “Incident reporting has come to stay” building on the categories entitled perceived advantages, observed changes and value of the IR system, and “Remaining challenges in incident reporting” including the categories entitled need for action, encouraged learning, continuous culture improvement, IR system development and proper use of IR.

**Conclusions:**

After 10 years, the practice of IR is widely accepted in the selected setting. IR has helped to put patient safety on the agenda, and a cultural change towards no blame has been observed. The informants suggest an increased focus on action, and further development of the tools for reporting and handling incidents.

**Electronic supplementary material:**

The online version of this article (10.1186/s12913-018-2876-5) contains supplementary material, which is available to authorized users.

## Background

Patient safety events in health care, in terms of incidents, near misses or unsafe conditions, have been discussed during the last decades, following the Institute of Medicine’s report To Err is Human in 1999 [1]. Incident reporting (IR) as a means to improve patient safety was one of the key recommendations in the report, and has been widely accepted. The purpose of IR is to identify safety hazards and, consequently, develop interventions to mitigate these hazards and reduce harm in health care. It has also been argued that a change in culture towards encouraging IR and focusing less on blame and personal responsibility is a path towards increased patient safety [2].

One problem in IR is that there might be confusion among staff regarding what should be reported. In the United States, only approximately 14% of all adverse events in hospital care are reported by staff, according to a report published by the inspector general of the Department of Health and Human Services in 2012 [3]. Explanations for this could be that the institution’s culture of safety is not conducive to reporting or that staff are not aware of what kind of events should be reported, which was confirmed in interviews with staff performed by Espin and colleagues, showing that there are considerable gaps between IR policy and practice [4]. Various incentives for IR has been tried and evaluated, including different reporting systems and the provision of feed-back [5, 6]. When Hewitt and colleagues compared how IR systems were used in two divisions at the same hospital they found a substantial variation in reporting, analysing, learning and feedback, which partly could be explained by how the system was introduced [7].

A barrier to IR is that healthcare providers do not prioritise reporting if they find the problem easily resolved [8]. They fix the problem and forget about it, and no learning occurs. It has also been shown that incidents are more likely to be reported if harm results [9].

Another great challenge in IR is that it is difficult to evaluate whether or not IR leads to improved patient safety, and the practice of IR has been discussed. Armitage and Chapman [10] conclude, referring to the curate’s egg, that it might be “good in parts”. Their advice is to focus on the reporting and analysing of severe incidents, which would hopefully lead to system change and improvement in patient safety. Pronovost and colleagues argue that reported incidents should be used to address and fix specific hazards in a clinical area or hospital, not to measure or monitor patient safety improvement [11]. When international patient safety experts were interviewed regarding their experiences of IR, a number of challenges could be identified, and one conclusion was that, to be effective, IR must be coupled with visible action [12].

The nature of IR and the paucity of quantifiable measures of its effectiveness demands diverse methods of assessment. Qualitative studies may reveal how IR is used and perceived in health care practice. A qualitative study among health care practitioners with experience of reporting and analysing incidents in health care showed that IR can contribute to the development and maintenance of risk awareness, but also revealed that learning from incident data is complex and difficult [13].

In Sweden all the county councils established web-based IR systems in the mid-2000s, putting Sweden in the frontline compared with other Nordic countries. IR is practiced in a similar way all over the country. A countrywide survey in 2011 showed that the practice of IR, together with root cause and risk analyses and legislation, were perceived to be the most important conditions for achieving the current level of patient safety [14].

After 10 years of practicing IR in Swedish health care we found it important to evaluate stakeholders perceptions of the practice. In the county council of Östergötland IR was first implemented in 2004, and there has been a conscious and persistent process supported by the authorities to sustain its use. Important stakeholders responsible for the implementation at department level are the heads of departments and the IR coordinators. These two groups represent different perspectives on practicing IR, why it is of interest to study their perceptions, their experiences over time, to what extent IR is found useful, and how they believe that IR can be improved. The aim of the present study was to explore the experiences of IR among heads of departments and IR coordinators in a Swedish health care setting where IR has been advocated and practiced for 10 years, to better understand their views of the practice and their thoughts regarding future application in order to enhance patient safety.

## Methods

### Study setting and design

Data collection was performed in Östergötland County, Sweden, which has approximately 450,000 inhabitants and is considered representative of Sweden in terms of age distribution, employment rates and economy. Health care in Sweden is publicly funded and is provided by the 21 county councils. The county councils are obliged to report incidents in health care [15]. The electronic IR system Synergy (developed by Det Norske Veritas Germanischer Lloyd AS, Norway) was implemented in Östergötland in 2004 and is still in use. In Synergy, all types of near misses and incidents can be reported by individual staff members or by the heads of departments. A brief description of the incident is entered, together with additional data based on pre-fixed multiple-choice responses. At each department there is usually one coordinator who receive the information and perform a brief investigation (e.g. discussion with involved individuals), classify the case, and hand it over to a superior who decide on further investigation or actions for improvement. The heads of departments are ultimately responsible for the reporting.

The County Council of Östergötland includes three hospitals and 41 primary health care centres. The present study focuses on IR in hospital care, where incidents occur frequently and are often severe to their nature [16]. Primary care was thus excluded, and in order to maintain homogeneity only somatic care was included. The study includes two groups of informants from the three hospitals: heads of departments and IR coordinators. The reason to include these two groups was that the heads of departments are ultimately responsible for patient safety including IR at their departments, and the IR coordinators are the ones who are in charge of the local performance of IR.

The study was performed with a qualitative design applying individual interviews and focus group discussions and was guided by the COREQ checklist (Additional file 1).

### Recruitment

Ten of the 45 eligible heads of departments at the three hospitals were invited to be interviewed. Selection was purposive so that the sample would cover different specialties, backgrounds (medical doctors and nurses) and levels of experience, and include both men and women. If the invited person did not agree to participate, another person with similar characteristics was invited. Invitations were sent by e-mail and, when a positive response was received, the head of department (referred to as the manager) was contacted again by e-mail or telephone to set up a time for the interview.

At the three hospitals, there are approximately 150 IR coordinators. All those were contacted by e-mail with an invitation to participate in a focus group discussion regarding IR. Two focus group discussions were held with the first coordinators who accepted the invitation.

### Data collection

A semi-structured interview guide was prepared in advance and the same guide was used for both managers and IR coordinators (Additional file 2). The questions concerned perceptions and opinions about IR, how IR affects health care and patient safety, opinions about the local IR system and suggestions for improvement based on experiences. Two pilot interviews with managers, not included in the analysis, took place and resulted in minor changes in the interview guide.

Of the ten managers who agreed to participate, one did not show up for the interview due to other urgent matters. This happened twice, and a decision was made to exclude this informant and proceed with nine individual interviews.

The individual interviews were performed by the author AG between February and June 2014. They were digitally recorded and transcribed verbatim. The interviews took place in the manager’s office and lasted between 17 and 42 min. Seven of the managers were physicians and two were nurses, five were male and seven had more than 5 years of experience in their current position.

Two focus group discussions were performed at the two largest hospitals, one with five participants and the other with four participants. All the participants were female nurses or midwives. The discussions were moderated by AG and SC took part as an observer. After each interview, AG and SC had a brief talk to discuss their impressions, as suggested by Krueger [17]. The discussions were digitally recorded and transcribed verbatim. Focus group discussions took place in May 2014; one lasted 53 min, the other 64 min.

### Data analysis

Data from the interviews and from the focus groups were analysed using qualitative content analysis [18]. Meaning units with essential content were inductively identified and labelled with codes. Codes were then organized into sub-categories, which were clustered into categories. The categories build on the manifest content from the data. Finally, overarching themes were identified as a way to link the underlying meanings, i.e. the latent content, together as described in the literature [18]. The initial analysis was performed by SC, after that discussions were held between all the three authors until consensus was reached. The computer-based analysis program NVivo 10 was used as an aid for the analysis. The quotes were translated from Swedish to English by the first author, and were discussed among all the authors. When uncertainties occurred these were discussed and solved by consulting a native English professional proof-reader. Finally, the quotes were re-translated into Swedish by a person outside the author group, to verify that no important content had been lost.

## Results

Eight categories, based on the manifest content, emerged from the inductive analysis of the interview data, and these categories could be allocated to two main themes reflecting the underlying, latent content. The theme “Incident reporting has come to stay” describes the latent content of the categories: perceived advantages, observed changes over time and value of the IR system. The theme “Remaining challenges in incident reporting” reflects the latent content from the categories: need for action, encouraged learning, continuous culture improvement, IR system development and proper use of IR. The results are supported by quotes from the interviews and the focus group discussions. In the quotes, the authors’ explanations appear in brackets,. .. means hesitation and (.. .) means that some words have been left out. Managers are numbered M 1–9, and focus group members are numbered according to group and individual, for example, FGD 1:1.

### Incident reporting is here to stay

#### Perceived advantages

The informants described advantages with IR based on their experiences. They found IR important to help identify problem areas for patient safety and for quality improvement. Another advantage mentioned was that IR helps to put patient safety on the agenda. As a result of the information from the IR coordinators at staff meetings, patient safety is discussed on a regular basis. It was also stated that IR is essential in health care as a means to assess quality.

Incident reporting has provided insight into what areas are problem areas, in our case for example medication issues, handling referrals, transcription of recorded documentation and communication with other departments (M 1)

Practicing IR has helped us to identify things that are actually a problem (FGD 1:3)

It is a kind of reminder to everyone, that this is high on the agenda, so I think it does make a difference (M 8)

We did not, we would not have worked with improvement in the way that we do if we had not practiced IR (FGD 1:4)

There’s an incredible interest. I mean, from the colleagues, when we have staff meetings (…) and it is time for NN (the IR coordinator) to present reported incidents, people are so excited, they find it so interesting (FGD 2:5).

There were statements from the managers about IR being of low value and not adding anything to patient safety. The informants who made these comments, however, were not entirely negative; they also had a number of positive experiences, showing that they still saw advantages with IR. This indicates that there is still ambiguity in some groups, while the general perception is positive.

I doubt that it is worth the time invested…the IR system helps us to find the flaws… to strengthen us. See, there was a positive connection too, good! (M 2)

#### Observed changes over time

The informants described a number of positive changes in attitudes during the past 10 years. From a rather reluctant attitude to IR, staff are now eager to observe and report incidents. Doctors still report incidents less frequently than other groups, but reporting is increasing over time and reports from doctors are considered to have a high level of relevance for patient safety.

The informants have also observed a change in culture from punishment, or a focus on who made the mistake, to a system perspective and more of a no blame culture*.*

Things are changing. It has become perfectly normal to report what is observed (FGD 2:4)

There was the attitude that, ‘if you do not do as your told, I will report that as an incident’ , I mean using the system to punish someone, and that has disappeared (M 2)

It is now understood that what we look for are system failures, not individuals (FGD 2:3)

Both managers and IR coordinators mentioned changes in practice that they judged to be important for improved patient safety and resulted from the reporting of incidents.

Then they assailed us with incident reports which led to… we had to do something about it, so we bought new equipment and after that it has worked out fine (M 5)

We did actually find a solution that we believe will decrease the problem (FGD 1:3)

#### An electronic system for IR is of great value

The local electronic IR system was considered to be of great value. The informants expressed how they feel that it is necessary to have a similar system to provide a structure for IR. They described their experience from previous handling of reported incidents in discussion groups, which was perceived as inefficient. They found it positive that the system is accessible to all staff members and saw how the system supports activities for patient safety improvement. The system was found to contribute to making patient safety work visible to staff. The IR coordinators also mentioned that the IR system provides external input, as reports from other clinics also appear, which was considered very positive.

The good thing with this IR system, in my opinion, is that it provides a kind of structure (M 9)

… and, it’s like everyone has access to the IR system, and everyone can report. So it does, you know, not depend on the channels (M 4)

It encourages you to initiate a process of change… you have to deal with it, you cannot pretend that the problem does not exist (FGD 1:3)

Sometimes it is of great value to get external input, because it highlights problems that we do not see ourselves (FGD 1:4)

### Remaining challenges in incident reporting

Despite all the positive perceptions that were expressed by the informants regarding IR, it became obvious that they still see a number of challenges that need to be overcome to increase value for money in IR.

#### Need for action

The managers expressed a lack of visible outcomes, and perceived a need for more measures to be taken based on the incidents reported, whereas the IR coordinators expressed the challenge of suggesting effective measures. If no results can be registered, motivation to continue with the reporting will decrease. The feedback mechanisms need to be improved so that the reporter or the reporting unit is informed about the results. The informants also stated that they would like to spend less time on reporting incidents and more time on actually implementing change to avoid repetition or similar incidents.

If we see benefits from reporting, then we will be motivated to report, but if we see that reports do not lead to improvements, the next time we will not report (M 7)

Then it is about the measure to be taken, and that is my responsibility, to be… imaginative about that. That’s the tricky part (FGD 2:1)

It is important that you have enough time to continue to handle the reported incident, from the reporting phase into reality, do something that changes how we work, that is what’s most important (M 4)

Among the managers, there were also statements showing a frustration regarding reporting and increased administration in health care, which, according to the informants, might lead to decreased patient safety.

We have all these web-based systems for reporting, to quantify, to do this and that, to measure that we do things within the stipulated time frames and so on, and it has not a damn thing to do with quality, safety, or anything (M 2)

#### Encouraged learning

Outcomes from IR were described by the managers as an important source for learning. Based on patterns emerging from IR, changes can be initiated. It would be of value to receive information about incidents and measures taken in other locations, local as well as national.

It is important for us to see… Does the reporting of incidents result in changes? (M 3)

… and how to learn from what went wrong, from the misses, instead of just stating that something went wrong again (M 7)

I always say: How do they handle these things in other places, and what can you learn from them? (M 6)

#### Continuous culture improvement

Patient safety culture has improved, but there are still challenges regarding culture. Some staff members have negative attitudes to IR; they interpret a reported incidence as critique, or they claim that reporting incidents will result in less time for patient work. Another concern is the use of IR as a means to punish individuals, in particular from other professional groups, by mentioning names and describing mistakes. This has decreased, but is still a problem. Under reporting was also mentioned, and there seems to be a need for continuous efforts to increase reporting.

It is almost like, almost like war you know, because some people, some people feel that you should not criticize each other (M 9)

Sometimes, instead of discussing face-to-face with the physician who did something wrong, they make it an incident report, you know (M 8)

Instead of handling the problem, you blame it on someone else (FGD 2:4)

I think the big problem is that a lot of things that should be reported are still not being reported (M 8)

A lack of cooperation between units regarding IR was described by the informants. They believe that increased dialogue would help to solve problems that are now handled by IR coordinators back and forth.

And then I’m back to this, this cooperation regarding incidents, which could be handled in a totally different way (M 9)

We send the reports back and forth… I really would like to have a forum where we could work on them together… (FGD 2:1)

#### IR system development

Several suggestions were made regarding the IR system in use. Making it more user friendly would mean that less time would be spent on reporting, and that staff members who now hesitate to report might start to do so. Some of the IR coordinators found the IR system difficult to handle, whereas others had no objections regarding the system.

Easier to fill out (…) so that there are less barriers and you will see more people reporting (M 6)

The reporting system itself is not very smooth, which is a disadvantage when you are trying to encourage more people to use it (M 7)

Should we really keep on dealing with all those papers in the system, just because we are told to? I’m not so sure about that, I’m not so sure about it (M 9)

If it was user friendly, we would have more, more (safety) problems would be made visible (FGD 1:4)

The option to get overviews from the system was requested, indicating that some of the informants are not fully aware of the functions that are already available or that they find the system too complicated to use.

#### Proper use of IR

There was also a suggestion to sort the reports into two categories: one for cases that are less severe and could be handled and fed back on a group level, another for severe cases that require individual handling and specific measures. The managers believed that this would also increase the motivation to continue reporting. The IR coordinators saw advantages with handling all sorts of IRs in the same system, and were reluctant to create parallel systems, fearing that reporting might decrease.

Some could be more routinely dispatched, could be grouped and dealt with on an overall level; that would be enough, instead of writing individual responses and do this and do that… (M 2)

You could turn it around a bit and maybe make it simpler, and then try to focus perhaps on… on bigger issues (M 9)

Everyday issues that are quite trivial are not a matter for the IR system, but… you cannot let it pass… but it should be handled in a much easier way (FGD 1:4)

The informants also expressed that some issues should not be handled in the IR system. If there is a communication problem, they believe that it is better to have an open discussion than to hide behind an incident report. Other less severe cases are also thought to be better solved in dialogue than being reported as an incident.

Then we have these… communication issues, personal issues between staff members, that are better solved in a direct communication, where we ask the staff members involved to sit down and talk instead of involving the IR system as a third party (M 1)

Some issues do not reach the level where they…you rather prefer to handle them in dialogue (M 9)

(aggravation among colleagues) should not be thrashed out in an IR system (FGD 1:2).

To be used in an efficient way, IR must be given reasonable resources. Too little investment, be it in terms of personnel or budget, will not lead to positive outcomes. Managers believe that IR can help to increase patient safety only if resources are allocated. But there is also a limit where it is not wise to invest money if risks are low. IR coordinators believe that more time would allow them to work more efficiently with patient safety issues based on IR.

And that is a way to make it safer for the patients, building structures all the time… but it also consumes resources (M 6)

They have time specifically allocated for this, which is also absolutely necessary (M 9)

How much to invest in order to avoid a risk? A risk that may not lead to a cost… you have to consider that not all risks that are identified will cause a mistake that renders a cost in health care (M 1)

You could work more on measures to be taken, and follow up information from statistics (if more time was allocated) (FGD 2:5)

## Discussion

The main finding from the study is that, after 10 years, IR is accepted and appreciated by representatives from the two groups of stakeholders. The managers find IR helpful in identifying problems relevant to patient safety, and have no intention to abandon the practice. The IR coordinators report a number of positive changes experienced over time, and can also verify that IR is widely accepted among all staff groups. This was interpreted as IR having come to stay, which could be a result from the explicit intention pronounced by the authorities, at both national and local level, to maintain IR despite initial reluctance. The informants also expressed a number of challenges in IR, in particular regarding observable outcomes, and continuously fostering a culture of reporting but without blame. At local level, developing the IR system to make it more user friendly and efficient is considered a challenge. The general positive perception of IR is in contrast to some of the criticism regarding IR that can be seen in the literature [10, 19], and proposals to stop reporting incidents [20].

One important finding from the study is that managers as well as IR coordinators perceive that IR helps to put patient safety on the agenda. This might be one of the most important functions of IR, given the perhaps oversized role of formal metrics in quality and safety [21].

An interesting, and somehow unexpected, finding was that there were very few discrepancies between the two groups included. In most of the issues they share the same view, but in some aspects the heads of departments differed from their IR coordinator colleagues, e.g. when it comes to increased paper work, and regarding learning from IR, discussed later. The perceived increase in paper work could be explained by the fact that the IR coordinators have allocated time for the issue, while the heads of departments are expected to do this as part of their ordinary work.

Cultural factors influencing IR have been discussed over the years. Culture, in this case, should be understood as a set of assumptions, values and norms shared by staff at the health care unit [22]. A more positive safety culture has been found to be associated with higher reporting rates [23].

Wachter and Pronovost [24] discuss patient safety culture in terms of no blame versus accountability, and state that what we need is a just culture, with a balance between no blame and individual accountability. The idea of pointing out the individual who failed, but without focus on blame, was mentioned also among the heads of departments in the present study. Khatri and colleagues [2] suggest the employment of a commitment-based management philosophy to support moving from a culture of blame towards a just culture, and argue that this could be a means to decrease the number of medical errors. In our study, it seems that no blame is the aim, in contrast to the previous focus on who committed the error. Our informants have witnessed a cultural change towards a no blame culture, but despite the observed change, they realize that there is still a long way to go. There are several examples, in particular mentioned by the heads of departments, of a culture where blame is still the focus.

In parallel with a movement away from a blame culture, an increased focus on learning from IR has been advocated [2], and was also an important challenge mentioned by the managers in our study. A “learning frame” has been suggested as an enabler to reporting as it allows depersonalization of IR [25]. The complexities involved and the difficulties in learning from IR data, however, should not be underestimated [15]. A prerequisite for learning from IR is, according to Maharajan, that information from reporting is fed back [26]. Benn and colleagues talk about closing the “safety feedback loop”, suggesting that better ways to feed information back will result in improved action based on IR [27].

This leads to the final important category mentioned by our informants: the lack of visible action based on IR. This was mentioned almost 10 years ago by Armitage and Chapman [10] suggesting that an analysis of evidence of action taken following IR would reveal low levels of activity. Patient safety experts interviewed in a qualitative study similarly concluded that IR in the future must be coupled with visible action, and that IR must be taken seriously by health care authorities [12]. Merely reporting does not improve patient safety, and if no results are fed back to the reporters, the practice will probably fade out over time. Action planning, however, presents several challenges as there historically has been a lack of tools to support the task, which is reflected on by Pham and colleagues [28], suggesting new approaches. When Macrae [29] discusses problems with incident reporting one important insight is that too much is collected and too little is done. Action based on IR, as called for by both managers and IR coordinators in the present study seems to be crucial.

Another suggestion made by our informants was to develop the IR system to make it more user friendly. When similar systems have been studied, a user-centered design has earlier been shown to be of importance [30], while inadequate reporting tools have been identified as barriers to IR [31]. The informants in the present study also called for a way to distinguish between severe and less severe but frequent incidents, in order to improve IR and increase motivation to continue reporting. Less severe incidents need to be accounted for, but a minimum of time should be spent on each report. More research is needed to develop efficient ways to handle these different types of events.

Implications from this study are that an explicit focus on IR results in positive changes over time, but 10 years is a too short period to reach the goal in all aspects. Time and resources should continuously be allocated for IR, and a shift from reporting to action is necessary. The development of tools in order to facilitate reporting, analysis and action is crucial, and distinguishing severe events from less severe but frequent events in reporting as well as in action may be an important change. If the mentioned conditions can be reached, IR has a higher potential to increase patient safety, and hopefully result in better value for money.

### Methodological considerations

As with all qualitative research, this study is limited regarding its relevance and generalizability to other settings and populations. All the informants were recruited from one region in Sweden, and may not be representative of the whole country. Among the heads of departments the majority were physicians, while the focus groups consisted of nurses and midwifes. The professional background may have influenced the informants, but considering that there were only few discrepancies between the two groups, it seems not to have had substantial importance.

Trustworthiness can also be discussed in terms of credibility, which was obtained by repeated discussions among the authors during the analysis process, and by the description of the different steps in the analysis. Regarding dependability, it was considered a strength that the author who performed the individual interviews and moderated the focus groups (AG) is herself a physician and has also served as an IR coordinator. The interviews as well as the focus group discussions took place with an air of confidence and security, and it seems that the informants did not hesitate to express their thoughts.

## Conclusions

After 10 years of continuous encouragement to implement and maintain IR, the practice is widely accepted in the selected setting. IR is considered to be valuable by both managers and IR coordinators, representing different perspectives on the issue. IR has helped to put patient safety on the agenda, and a cultural change towards no blame has been observed. The informants suggest an increased focus on action, and the development of tools to facilitate reporting and action based on the severity of the incidents.

## Additional files

Additional file 1: COREQ Checklist. (DOCX 16 kb)

Additional file 2: Interview Guide. (DOCX 12 kb)

## Abbreviations

FGD: Focus group discussion
IR: Incident reporting
