# Supplementary material for: Experiences from ten years of incident reporting in health care: a qualitative study among department managers and coordinators
Source: BMC Health Serv Res. 2018 Feb 14;18:113. doi: 10.1186/s12913-018-2876-5 (PMC5813432; doi:10.1186/s12913-018-2876-5)
Supplement: Supplementary file 1 — COREQ Checklist. (DOCX 16 kb) [file 12913_2018_2876_MOESM1_ESM.docx]

**Consolidated criteria for reporting qualitative studies (COREQ): 32-item checklist**

| **No. Item** | **Description** | **Reported on Page #** |
| --- | --- | --- |
| **Domain 1: Research team and reﬂexivity** |  |  |
| *Personal Characteristics* |  |  |
| 1. Interviewer/facilitator | Author AG conducted the interviews/focus groups | Methods, p 7 |
| 2. Credentials | Two authors were PhDs one author MD | Author list, title page |
| 3. Occupation | Not considered relevant. | NA |
| 4. Gender | Not considered relevant. | NA |
| 5. Experience and training | The interviewer is an MD, physician with experience in the field under observation. | Methodol. considerations, p 18 |
| *Relationship with participants* |  |  |
| 6. Relationship established | No relationship was established in advance, however, some knowledge about the interviewer cannot be ruled out | Not reported |
| 7. Participant knowledge of the interviewer | Information was provided in connection with the interviews. | Interview guide |
| 8. Interviewer characteristics | The interviewer is described in the manuscript. | Methodol. considerations, p 18 |
| **Domain 2: study design** |  |  |
| *Theoretical framework* |  |  |
| 9. Methodological orientation and Theory | Qualitative content analysis was applied | Methods, p 7 |
| *Participant selection* |  |  |
| 10. Sampling | Purposive sampling was applied | Methods, p 6 |
| 11. Method of approach | Participants were invited by e-mail and interviews were conducted face-to-face | Methods, p 6 |
| 12. Sample size | Nine individual interviews, focus groups with another nine individuals. | Methods, p 6 |
| 13. Non-participation | Based on inclusion criteria defined, the first who accepted were included . | Methods, p 6 |
| *Setting* |  |  |
| 14. Setting of data collection | Data was collected at the informants workplace. | Methods, p 7 |
| 15. Presence of non-participants | No non-participants were present | Not reported |
| 16. Description of sample | Demographic data is reported on a general level, to avoid identification of informants. | Methods, p 7 |
| *Data collection* |  |  |
| 17. Interview guide | The interview guide is attached. | Supplementary material |
| 18. Repeat interviews | No repeat interviews were conducted | NA |
| 19. Audio/visual recording | Audio recording was used to collect the data | Methods, p 7 |
| 20. Field notes | No ﬁeld notes were made | NA |
| 21. Duration | Duration of the interviews and focus groups is reported. | Methods, p 7 |
| 22. Data saturation | Data saturation is not applicable when content analysis is used. | NA |
| 23. Transcripts returned | Transcripts were not returned to participants for comment and/or correction | NA |
| **Domain 3: analysis and ﬁndings** |  |  |
| *Data analysis* |  |  |
| 24. Number of data coders | One initial coder, all coding was discussed among the authors. | Methods, p 7 |
| 25. Description of the coding tree | The coding tree is not included in the manuscript, only themes and categories are presented. | NA |
| 26. Derivation of themes | Themes were derived from the data | Methods, p 7 |
| 27. Software | NVivo was used to manage the data | Methods p 7 |
| 28. Participant checking | Participants did not provide feedback on the ﬁndings | NA |
| *Reporting* |  |  |
| 29. Quotations presented | Participant quotations were presented to illustrate the themes/ﬁndings, each quotation identiﬁed using participant number | Results |
| 30. Data and ﬁndings consistent | There is consistency between the data presented and the ﬁndings | Results |
| 31. Clarity of major themes | Major themes are clearly presented in the ﬁndings | Results |
| 32. Clarity of minor themes | Categories and themes are described and discussed | Results / Discussion |
