# Supplementary material for: Experiences from ten years of incident reporting in health care: a qualitative study among department managers and coordinators
Source: BMC Health Serv Res. 2018 Feb 14;18:113. doi: 10.1186/s12913-018-2876-5 (PMC5813432; doi:10.1186/s12913-018-2876-5)
Supplement: Supplementary file 2 — Interview Guide. (DOCX 12 kb) [file 12913_2018_2876_MOESM2_ESM.docx]

Interview guide

A. General information about purpose, ethical issues, etcetera.

B. Main interview questions:

1. How, in your opinion, is patient safety influenced by incident reporting…

… in general?

… at your department?

Could you give me examples?

2. How are health care activities i.e. the daily work, influenced by the existing demand to report incidents…

… in general?

… at your department?

3. What are the outcomes from incident reporting?

Based on your own experiences… (positive – negative)

Is there room for improvement regarding outcomes? Suggestions…

4. What is your opinion about Synergi (the IR-reporting system)?

Why do you think we have an electronic IR system?

Is the technical solution appropriate?

Satisfactory?

Time consuming?

5. Do you have any suggestions for improvement regarding incident reporting in health care?

How IR is organized locally…

Legislation…

Technically (the electronic IR system)…

Other…

C. Concluding remarks.

*Probes are used only if the answers do not emerge without articulating the questions.*
